# Mesothelial cyst derived from chest wall pleura growing after thoracic surgery: a case report

**DOI:** 10.1186/s13256-018-1944-0

**Published:** 2019-01-06

**Authors:** Hiroyasu Matsuoka, Hirochika Matsubara, Aya Sugimura, Tsuyoshi Uchida, Tomofumi Ichihara, Tadao Nakazawa, Hiroyuki Nakajima

**Affiliations:** 1 Department of Surgery, Faculty of Medicine, University of Yamanashi, 1110 Shimokato, Chuo City, Yamanashi Japan; 2 Department of Pathology, Faculty of Medicine, University of Yamanashi, 1110 Shimokato, Chuo City, Yamanashi Japan

**Keywords:** Chest wall pleura, Extramediastinal mesothelial cyst, Peritoneal inclusion cyst, Postoperative

## Abstract

**Background:**

Intrathoracic mesothelial cysts almost always arise in the mediastinum, and extramediastinal mesothelial cysts are extremely rare. Here we describe a case of mesothelial cyst derived from the chest wall pleura growing after thoracic surgery.

**Case presentation:**

A 63-year-old Japanese woman was referred to our department. She had undergone total hysterectomy for cervical carcinoma and two lung wedge resections for metastatic lung cancer on the upper and lower lobes of her right lung and lower lobe of her left lung. After the thoracic surgery, an intrathoracic chest wall mass was found, which grew gradually. Computed tomography demonstrated a 2.0 × 1.8 cm low-density mass without contrast effect. Magnetic resonance imaging demonstrated a low-intensity mass in T1-weighted imaging and a high-intensity mass in T2-weighted imaging. Thoracoscopic excision of the mass was performed. The cystic mass was thought to be derived from her chest wall and was pathologically diagnosed as mesothelial cyst. Five years after the surgery, she has no evidence of recurrence of the cyst or cervical carcinoma.

**Conclusions:**

The genesis of extramediastinal mesothelial cysts may be related to inflammation. From this perspective, extramediastinal mesothelial cysts may have different characteristics from pericardial cysts and resemble peritoneal inclusion cysts. Although, extramediastinal mesothelial cysts are not established, their characteristics resemble peritoneal inclusion cysts; therefore, such interesting intrathoracic cysts should be carefully resected considering the risk.

## Background

Mesothelial cysts are derived from the pleura, pericardium, and peritoneum, structures that are composed of mesothelial epithelium. Intrathoracic mesothelial cysts almost always arise in the mediastinum and are derived from the pericardium; hence, they are often called pericardial cysts.

By contrast, mesothelial cysts arising from the extramediastinum are extremely rare. Hence, in a review of the English literature, only one report had described mesothelial cysts derived from the extramediastinum [1].

Here we describe a case of mesothelial cyst derived from chest wall pleura, which may embryologically differ from common mesothelial cysts, the so-called pericardial cysts that occur congenitally.

## Case presentation

A 63-year-old Japanese woman was referred to our department because of an abnormal shadow at the left side of her chest wall on computed tomography. She had undergone total hysterectomy and radiotherapy for cervical carcinoma 4 years prior. One year after the first surgery, three metastatic lung nodules appeared at the upper lobe of her right lung, the lower lobe of her right lung, and the lower lobe of her left lung. Wedge resection for upper and lower lobe of her right lung was initially performed via three-port thoracoscopic surgery. Then, wedge resection for the lower lobe of her left lung was performed via eighth intercostal single incisional thoracoscopic surgery. After the surgery, an intrathoracic chest wall mass developed which increased in size gradually. Her gynecologist introduced her to our department for surgical resection of the mass. Her family, including her parents and two sisters, had been healthy and had no inheritable diseases. She had no symptom, drug history, tobacco smoking history, or psychosocial history, and she was a social drinker. She had not received any medications since the mass developed and until admission to our hospital. She had undergone an operation three times as mentioned above and had been a carrier of type B hepatitis.

**Fig. 1:**
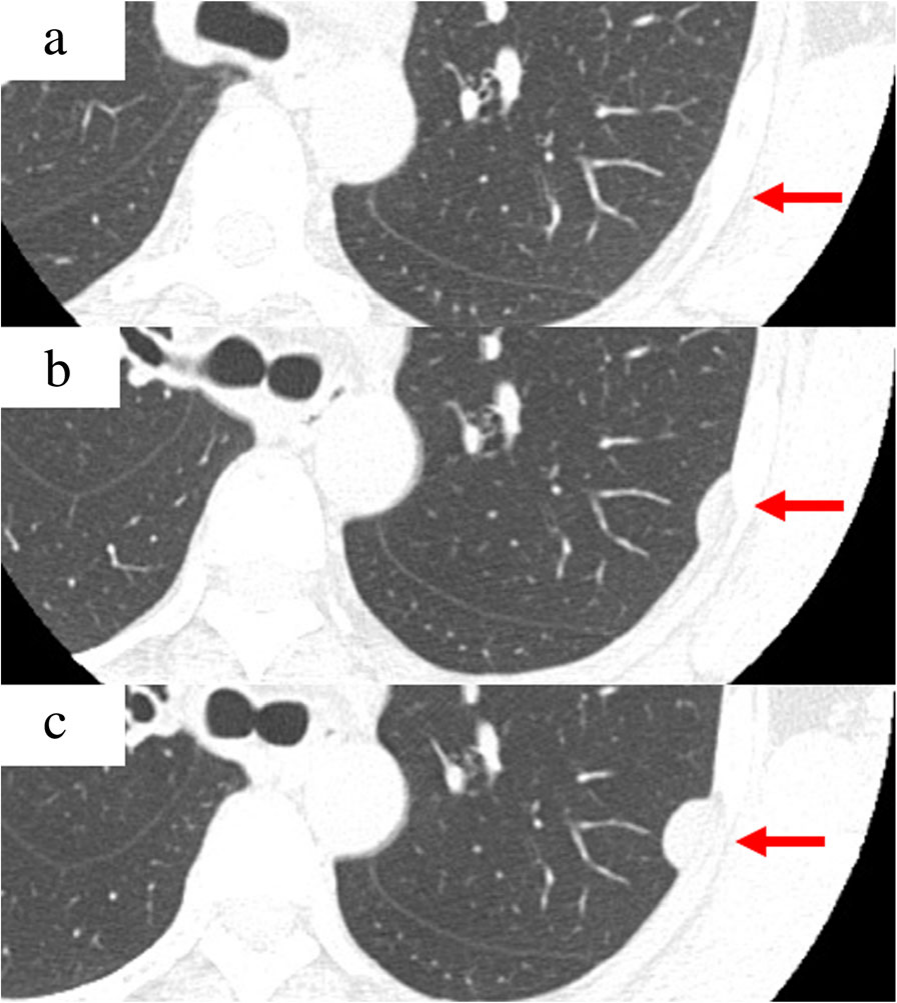
Computed tomography demonstrated a gradually increasing low-density mass (*arrows*) measuring 2.0 × 1.8 cm in diameter. There was no mass region at 6 months after the wedge resection of the lower lobe of the left lung (**a**). A flat region appeared 1 year after the wedge resection of the lower lobe of the left lung (**b**) with increasing size up to 2.0 × 1.8 cm at 2 months before the present surgery (**c**)

**Fig. 2:**
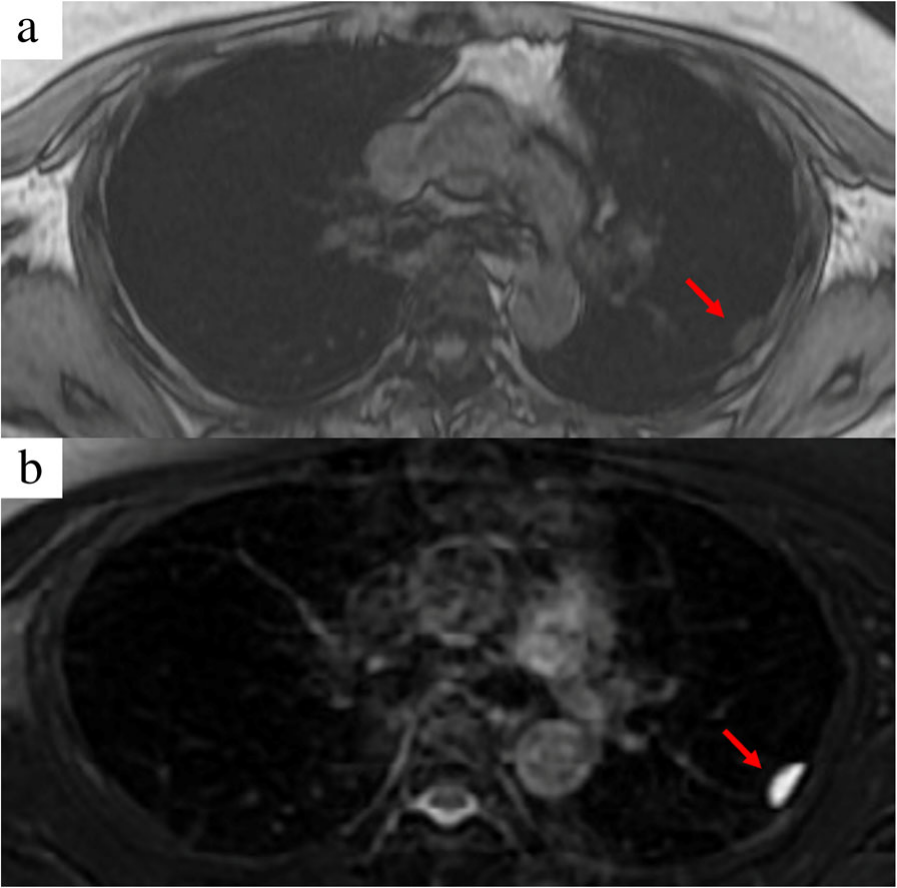
Magnetic resonance imaging demonstrated a low-intensity mass (*arrow*) in T1-weighted imaging (**a**) and high-intensity mass (*arrow*) in T2-weighted imaging (**b**)

After her admission to our department, her general condition was good, and there were three operative scars at both sides of her chest and lower abdomen. Her chest sounds were clear and there was no neurological abnormality. She was 151.1 centimeters tall and weighed 49.8 kilograms. Her heart rate was 77/minute, blood pressure was 135/87 mmHg, and body temperature was 36.1 °C. The laboratory findings were white blood cells 5.25 × 10^3^/μL, hemoglobin 12.7 g/dL, and platelets 156 × 10^3^/μL. A liver function test revealed: albumin 4.6 g/dL, aspartate aminotransferase 15 U/L, alanine aminotransferase 13 U/L, and total bilirubin 0.3 mg/dL. A renal function test revealed blood urea nitrogen 13.6 mg/dL and creatinine 0.79 mg/dL. An electrolyte test revealed sodium 143 mEq/L, potassium 3.8 mEq/L, and chlorine 106 mEq/L. A tumor marker test revealed carcinoembryonic antigen 3.4 ng/mL and squamous cell carcinoma antigen 0.80 ng/mL. Another test revealed positive reaction to type B hepatitis surface antigen and C-reactive protein < 0.1 mg/dL. Computed tomography demonstrated a gradually increasing low-density mass measuring 2.0 × 1.8 cm in diameter (Fig. 1). Magnetic resonance imaging demonstrated a low-intensity mass in T1-weighted imaging and a high-intensity mass in T2-weighted imaging (Fig. 2). The mass was thought to be a singular cyst; however, this type of cyst was rare and the mass was increasing. Therefore, dissemination of cervical carcinoma could not be excluded, and surgical removal of a part or tissue of the mass was performed.

**Fig. 3:**
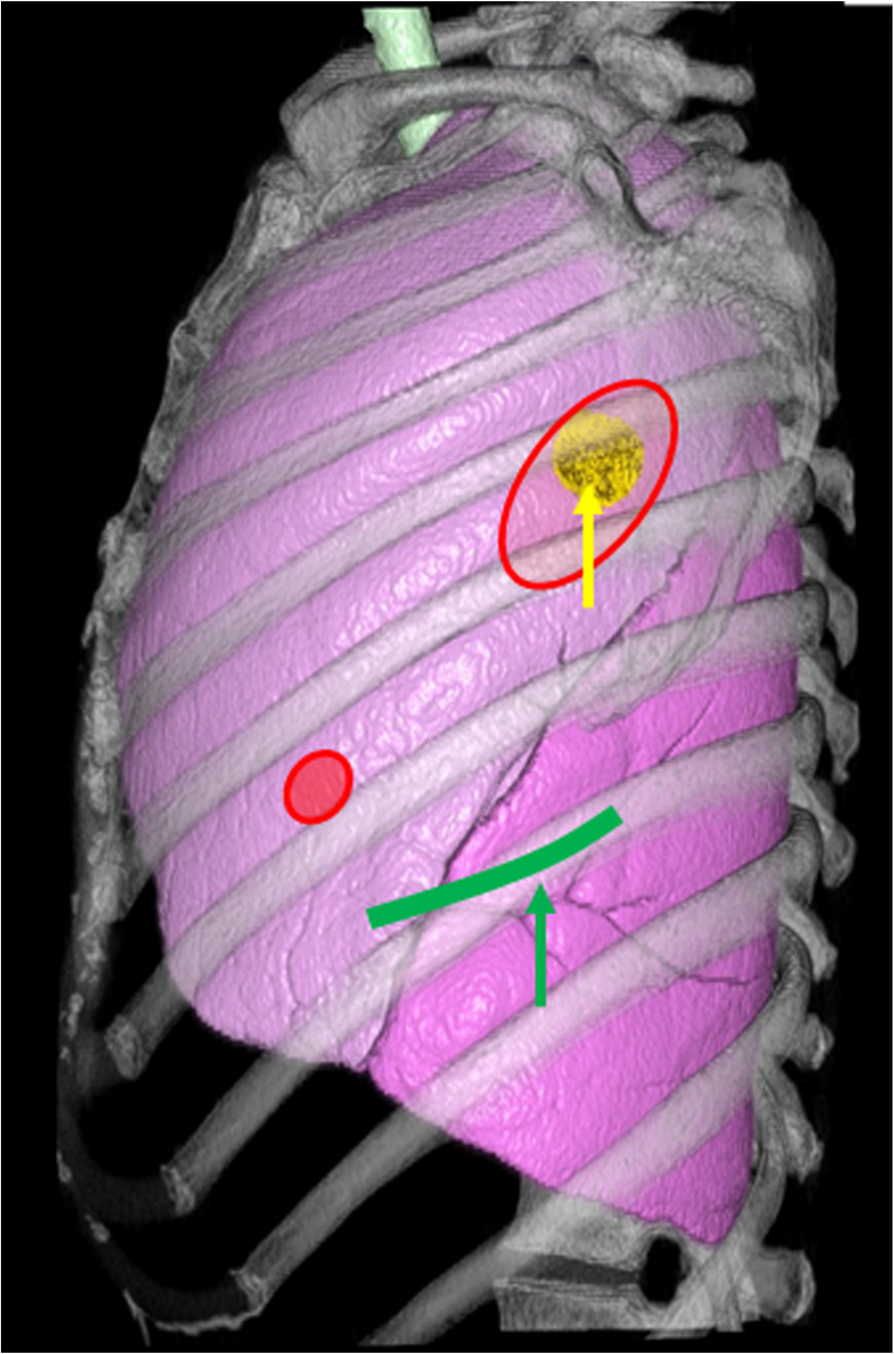
Three-dimensional image reconstructed from computed tomography with operative images. Thoracoscopic excision of the mass was done with two ports (*small red circle*, 3 mm port; *red large circle*, 2 cm access port). The cyst (*yellow arrow*) was far from the previous operative scar (*green curved line*). The lower lobe of the left lung had a fold (*green arrow*) that had been made from past wedge resection

In the right lateral position, thoracoscopic excision of the mass was done with two ports (3 mm and 2 cm access ports) by two general thoracic surgeons (Fig. 3). First the 3 mm port was set at the sixth intercostal space on the inframammary line. Most of her left lung was attached to her chest wall; therefore, the second port was set above the cyst and lysis of adhesions was done. After the lysis, the cystic mass was found adhering to the upper lobe of her left lung. The adhesion of the mass to her lung was not strong and could be separated without injury to the visceral pleura. Therefore, the mass was thought to derive from the chest wall pleura and was resected by adhesiolysis.

**Fig. 4:**
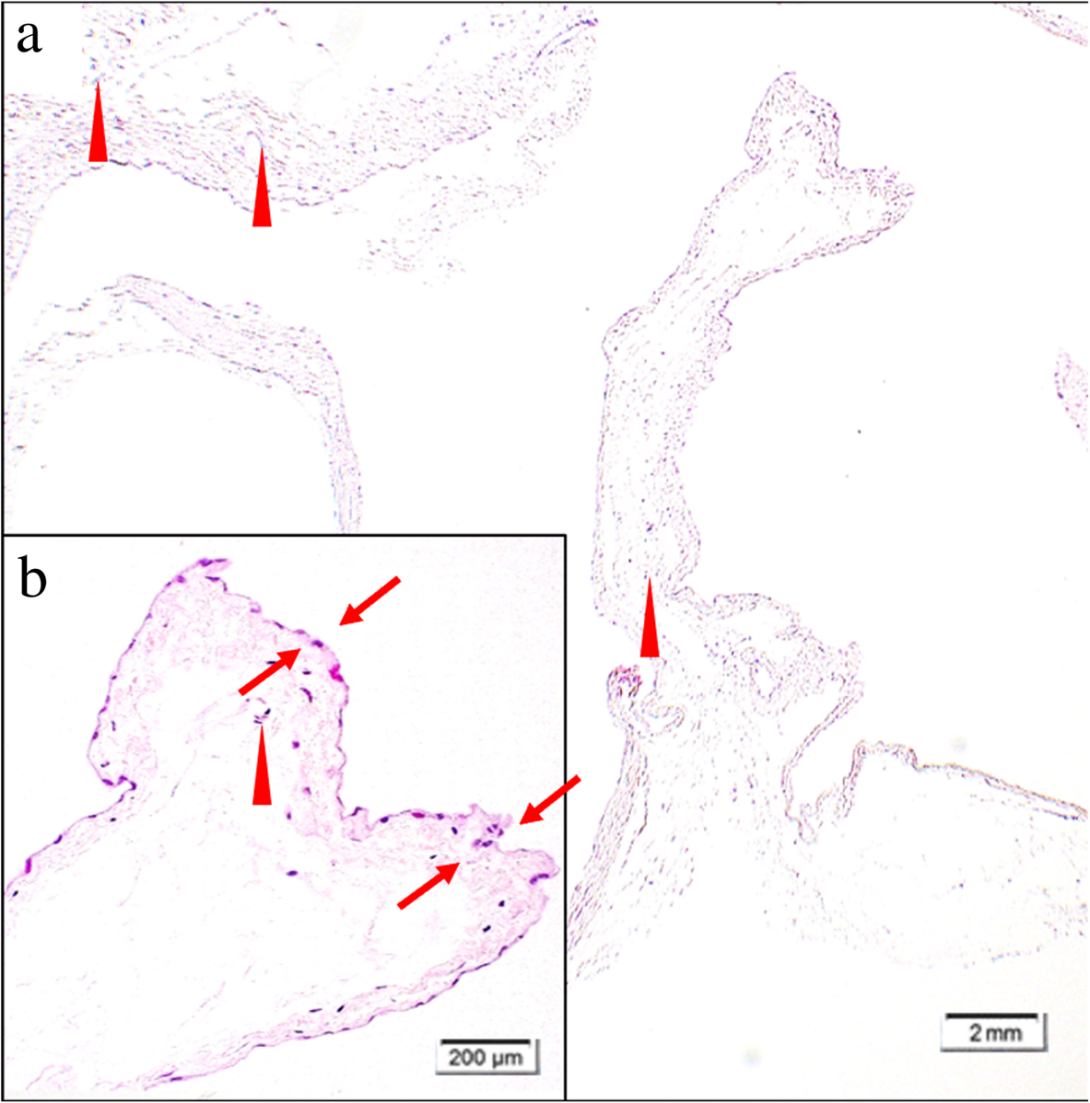
Microscopic findings (hematoxylin and eosin staining). Low-power field (**a**), High-power field (**b**). The cyst was lined with a single layer of cuboidal epithelium (*arrow*), and there were few infiltrations of inflammatory cells (*cone*)

**Fig. 5:**
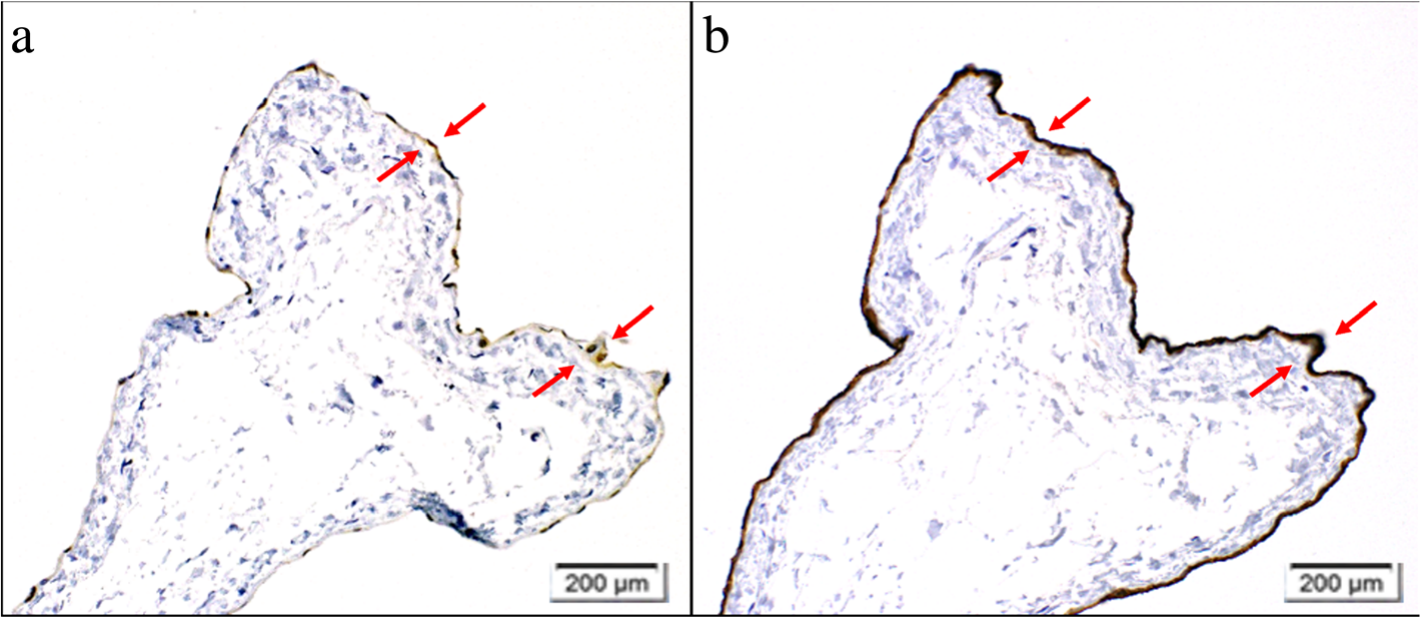
Immunohistochemical findings (calretinin and D2-40). Immunohistochemistry showed slightly positive staining of calretinin (**a**) and strong positive staining of D2-40 (**b**) in the cystic wall epithelium (*arrow*)

The mass was a unilocular cyst containing mucinous fluid. On microscopic examination, the cyst was lined with a single layer of cuboidal epithelium (Fig. 4); immunohistochemistry showed positive staining of calretinin and D2-40 (Fig. 5). Thus, the cyst was diagnosed as mesothelial cyst derived from the chest wall pleura. Five years after the surgery, our patient had no evidence of cyst or cervical carcinoma on computed tomography.

## Discussion and conclusions

Intrathoracic mesothelial cysts are not rare and most of these cysts arise in the pericardium congenitally. However, extramediastinal mesothelial cysts arising in the chest wall pleura are extremely rare. Our case report is the second report of such cysts, and the first report that mentions etiology associated with peritoneal mesothelial cysts. In our case, the cyst was acquired, and previous operative inflammation might have caused the cyst. An association between intrathoracic mesothelial cysts and inflammation is not well known and intrathoracic mesothelial cysts may be confused with other congenital cysts. Therefore, if there are more cases the same as ours, it is important to distinguish between congenital and acquired mesothelial cysts; comprehension of the etiology of acquired mesothelial cysts may lead to less invasive treatment.

Most intrathoracic mesothelial cysts are derived from the pericardium, especially from the cardiophrenic angle (92%) [2]. Normally, the cysts contain clear fluid, and the wall is covered by a single layer of mesothelial cell. Pericardial cysts are thought to be formed by a failure of fusion during pericardial sac formation [3]. Most atypically located mesothelial cysts were explained embryologically, except for cysts arising from the chest wall [4]. Therefore, the mechanism for extramediastinal mesothelial cysts may differ from the mechanism for pericardial cysts.

Monzen *et al.* reported that mesothelial cysts are derived from the parietal pleura [1]. In this case, there were mild inflammatory cell infiltrations to the wall of the cyst, and positron emission tomography integrated with computed tomography detected the cyst wall with a standardized uptake value of 2.0. In our case, although inflammatory cell infiltration was not clearly detected in the wall of the cyst, slow growth obscured pathological features and previous thoracic surgery might have caused the genesis of the cyst.

Although there are few reports of intrathoracic cysts caused by inflammation, there are many reports of peritoneal mesothelial cysts caused by inflammation, such as peritoneal inclusion cysts (PICs) [5, 6]. PICs usually occur in women of childbearing age, and most patients have a history of an abdominal operation or pelvic inflammatory disease, such as endometriosis. Therefore, inflammation has been thought to be the cause of PICs, and the cysts often adhere to the intraperitoneal organs. On pathological examination, PICs consist of a unilocular thin wall interlined by a single layer of mesothelial cells with watery to gelatinous contents [7]. Our patient had undergone a thoracic operation and had intrathoracic adhesion with the same pathological features. This suggests that the cyst may have occurred due to postoperative inflammation, and several intrathoracic mesothelial cysts may be caused by inflammation.

The prognosis of extramediastinal mesothelial cysts is unclear because they are very rare and there were few reports of recurrent pericardial cyst [8]. However, the prognostic information of PICs may be useful. There are some reports that PICs have a risk of recurrence [5, 6]. Excision of the cysts causes additional inflammation, and it seems to be the most common reason for recurrence. However, PICs have been reported to be a progressive disease and cause symptoms such as pain or organ oppression [6]. Although extramediastinal mesothelial cysts are not established, the characteristics resemble PICs; therefore, such interesting intrathoracic cysts should be carefully resected considering the characteristics of PICs. In addition, drainage is one of the optional therapies reported in PICs [5]. If there is no mass region on computed tomography or any symptoms, but there is a history of operation associated with a portion of the cyst, then drainage may be considered.

## Abbreviation

PICs: Peritoneal inclusion cysts
